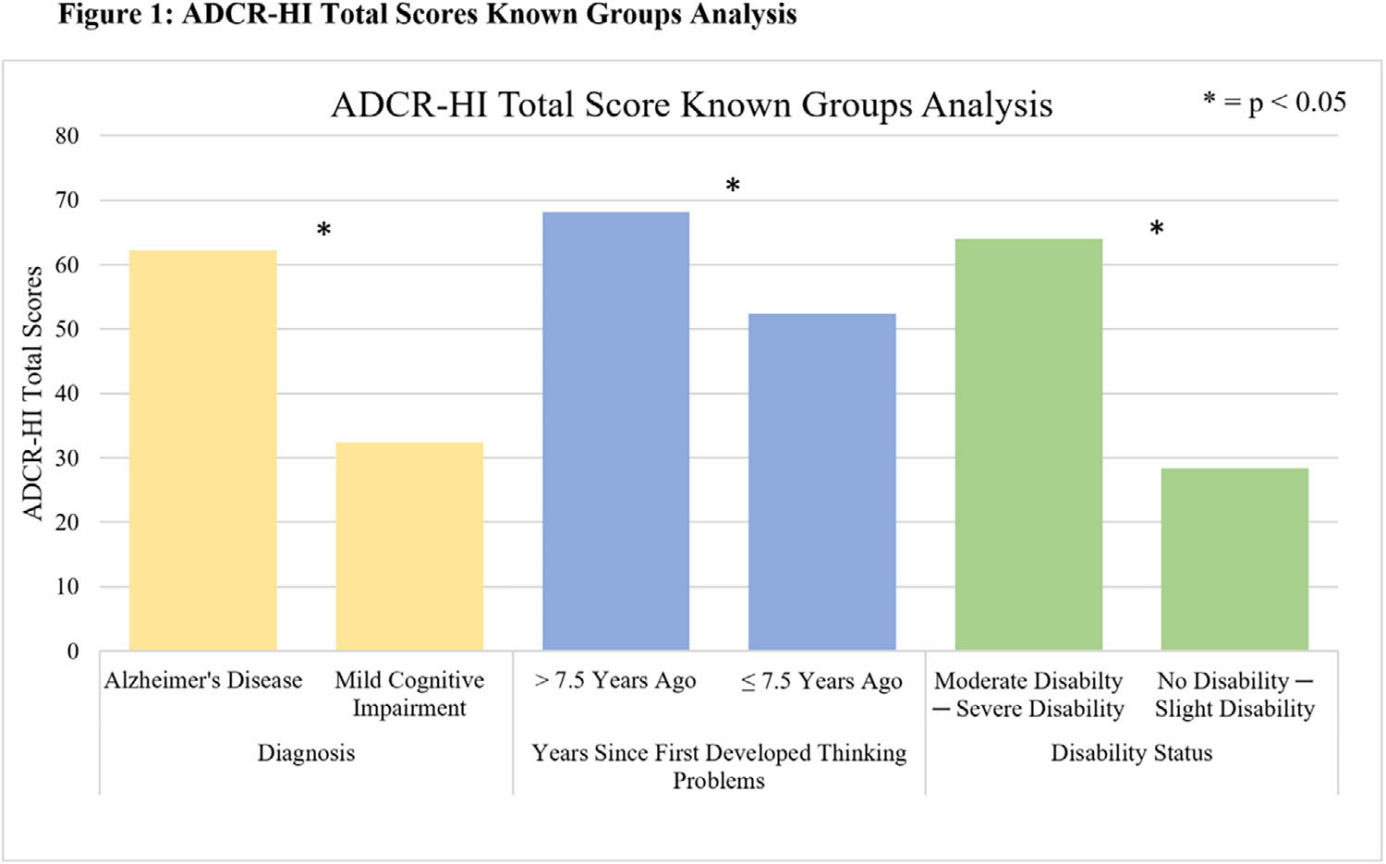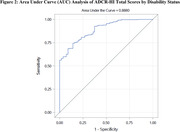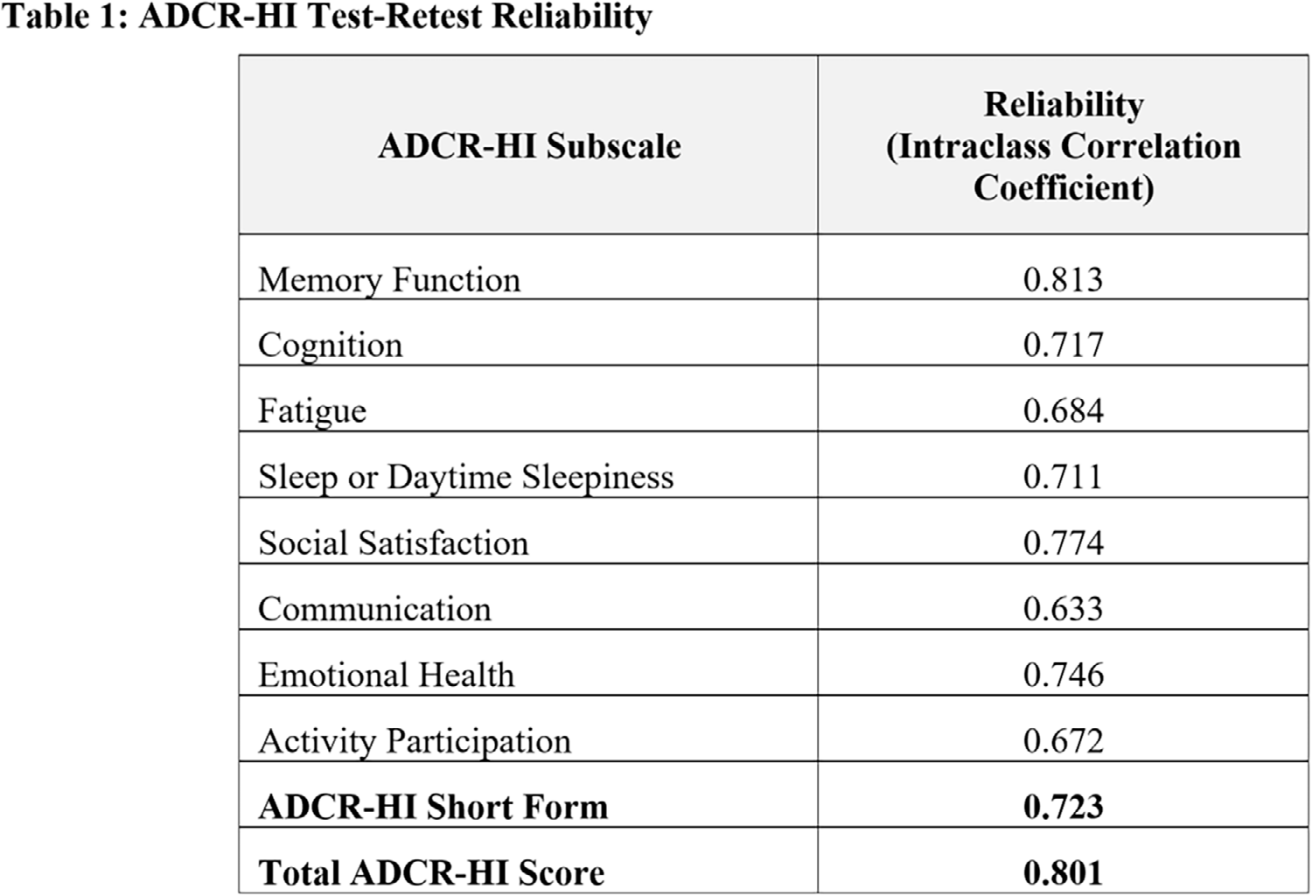# The development and validation of the Alzheimer’s Disease Caregiver Reported‐Health Index (ADCR‐HI): a disease‐specific caregiver‐reported outcome measure for use in clinical trials

**DOI:** 10.1002/alz.087655

**Published:** 2025-01-09

**Authors:** Christina Shupe, Jamison Seabury, Anika Varma, Jennifer Weinstein, Charlotte Engebrecht, Spencer Rosero, Charlotte Irwin, Nuran Dilek, Abigail Arky, Elizabeth Santos, Chad Heatwole

**Affiliations:** ^1^ Center for Health + Technology, University of Rochester Medical Center, Rochester, NY USA; ^2^ School of Medicine and Dentistry, University of Rochester, Rochester, NY USA; ^3^ Spencer Fox Eccles School of Medicine, University of Utah, Salt Lake City, UT USA; ^4^ University of Rochester Medical Center, Rochester, NY USA; ^5^ Des Moines University School of Medicine, Des Moines, IA USA; ^6^ Memory Care Program, University of Rochester Medical Center, Rochester, NY USA

## Abstract

**Background:**

In preparation for therapeutic trails involving patients with Alzheimer’s disease (AD) and mild cognitive impairment (MCI), there is a need for valid, disease‐specific caregiver‐reported outcome (CRO) measures capable of tracking symptomatic burden in response to therapy over time. CROs are useful tools in clinical trials for individuals with AD, MCI, and dementia who are unable to self‐report. In addition, CROs are accepted by the United States Food and Drug Administration to support regulatory claims. This research involved the development and validation of the Alzheimer’s Disease Caregiver Reported‐Health Index (ADCR‐HI): a regulatory grade, disease‐specific CRO for use in detecting symptomatic changes in individuals with AD, MCI, and dementia during therapeutic trials.

**Method:**

We conducted semi‐structured qualitative interviews and a cross‐sectional survey with caregivers to identify the prevalence and relative importance of symptoms experienced by individuals with AD, MCI, and dementia. We selected symptom questions for the ADCR‐HI based on their relevance and potential responsiveness to therapeutic intervention. We performed beta interviews with caregivers to determine the comprehensiveness, ease of use, and relevance of the instrument. Subsequently, we determined the reliability, known groups validity (**Figure 1**), and final internal consistency of the ADCR‐HI. We additionally conducted an area under curve (AUC) analysis using ADCR‐HI total scores (**Figure 2**).

**Result:**

Initial interviews were conducted with 15 caregivers of individuals with AD, MCI, or dementia. The cross‐sectional survey was completed by 324 caregivers. Beta interviews with 12 caregivers determined the instrument to be relevant and easy to use. The ADCR‐HI and its subscales displayed high test‐retest reliability over a 14 day period (n = 30) **(Table 1)**. Known groups analysis (**Figure 1**) demonstrated the ability of the ADCR‐HI to differentiate between groups with greater disease severity.

**Conclusion:**

The ADCR‐HI is a regulatory‐grade, reliable CRO comprised of eight subscales that comprehensively measure an individual’s symptomatic burden through the perspective of the caregiver. The ADCR‐HI is available to support therapeutic trials and drug‐labeling claims as a valid clinical tool.